# Physiological effect of iron status on patients with polycystic ovary syndrome in Basrah city

**DOI:** 10.5937/jomb0-39091a

**Published:** 2023-08-25

**Authors:** Dalal F. Al-Akabi, Hanadi A. Hafth

**Affiliations:** Al-Kunooze University College, Department of Medical Laboratory Techniques, Basrah, Iraq; Al-Kunooze University College, Dental Department, Basrah, Iraq

**Keywords:** iron, polycystic ovary syndrome, endocrine disorder, infertility, gvožđe, sindrom policističnih ovarijuma, endokrini poremećaj, neplodnost

## Abstract

Polycystic ovary syndrome (PCOS) is one of the most common gynecological diseases that affect the fertility in women in Basra governorate. The current study was designed in order to assess iron aberrations in PCOS patients by measuring the related parameters and their relationship with sex hormones in patients with PCOS. Serum samples were collected from 45 PCOS patients and 45 controls from a private women's clinic and were measured by ELISA in a private medical laboratory. The results showed a significant decrease in the level of hepcidin, transferrin and estradiol versus a significant increase in iron, ferritin, progesterone and testosterone. The current study showed a clear imbalance in the level of iron and its serum regulating parameters in in PCOS women, and there is an effective correlation between iron status and sex hormones.

## Abstract (sr)

Sindrom policističnih jajnika (PCOS) je jedno od najčešćih ginekoloških bolesti koje utiču na plodnost žena u provinciji Basra. Sadašnja studija je dizajnirana da proceni aberacije gvožđa kod pacijenata sa PCOS merenjem povezanih parametara i njihovog odnosa sa polnim hormonima kod pacijenata sa PCOS. Uzorci seruma su prikupljeni od 45 pacijenata sa PCOS-om i 45 kontrola iz privatne ženske klinike i mereni su ELISA testom u privatnoj medicinskoj laboratoriji. Rezultati su pokazali značajno smanjenje nivoa hepcidina, transferina i estradiola naspram značajnog povećanja gvožđa, feritina, progesterona i testosterona. Sadašnja studija je pokazala jasnu neravnotežu u nivou gvožđa i njegovih serumskih regulacionih parametara kod žena sa PCOS-om, i postoji efikasna korelacija između statusa gvožđa i polnih hormona.

## Introduction

Polycystic ovaries (PCOS) are a common endocrine disorder among women, where several factors contribute to its emergence, such as environmental and genetic factors, it is one of the most important causes of infertility PCOS associates with the appearance of acne and hirsutism and with age develops into metabolic disorders such as insulin resistance and cardiovascular diseases [1]. PCOS causes disturbances in the ovulation and an excess production of androgens, the pathogenesis responsible is not yet clear, and currently there is no definitive treatment, it is seems that visceral lipids cause an inflammatory response by releasing cytokines and interleukins, which affect the ovulation process [2]. PCOS causes an imbalance in the menstrual cycle and is accompanied by high weight and diabetes, currently the treatment of PCOS depends on the dominant symptoms only [3]. It has been proven that iron accumulation in the fatty tissue of PCOS patients is one of the causes of dysfunction in this tissue, and this appears especially in patients who suffer from obesity thus this confirm their metabolic disorder [4]. A close association between high ferritin levels and insulin resistance has been observed in obese PCOS patients, and this represents a clear manifestation of metabolic disorder [5]. Ferritin rises in PCOS patients, especially with abnormal sugar levels, due to high iron in the blood, this high iron is associated with several factors such as menstrual disorders, insulin resistance, and low hepcidin that increases iron absorption, it is not likely that elevated androgens may improve erythropoiesis due to transferrin level do not rise in PCOS women [6].The decreased hepcidin was observed in female patients and it showed a clear association with insulin resistance which may work together to increase the risk of PCOS [7]. Hepcidin impede iron absorption by the intestines, where the level of hepcidin decreases in PCOS, which leads to high iron levels and this is associated with the high androgens and insulin resistance that associated with PCOS, low hepcidin and high ferritin are also associated with oligoamenorrhea in PCOS patients [8]. Liver is responsible for the production of hepcidin, and any imbalance in the levels of this hormone is due to various reasons, such as inflammations, disruption of erythropoiesis process, or the interference of genetic factors, eventually that lead to abnormal levels of iron in the blood [9]. Iron absorption is controlled by hepcidin, and this ability is influenced by many factors such as sex hormones such as estrogen and progesterone [10]. Trans plays an important role in the metabolism of iron and its transfer to the cells of the body and also acts as a growth factor. The current study aims to study the physiological effect of iron alternation on PCOS patients and its relationship to some of serum parameters and some common clinical manifestations associated with these abnormalities.

## Materials and methods

### Study design and setting

The study was conducted in Basrah Governorate, Iraq, from May to September 2021. Serum samples were collected from 45 PCOS subjects and 45 healthy women during the luteal phase. Samples were collected from a private clinic under the supervision of a specialized gynecologist.

### Inclusion criteria and exclusion criteria

It was confirmed that all samples belong to women who do not suffer from chronic, hormonal, blood, inflammatory autoimmune diseases, hypertension, diabetes and drug interactions for at least the last 3 months that may affect the study parameters. Based on the questionnaire, PCOS patients were divided into groups according to (hirsutism, oligoamenorrhea, age, BMI, and infertility issues).

### Procedures

The levels of hormones were measured using special kits by ELISA.

### Hepcidin and Ferritin (SunLong Biotech/China) assay

The concentration of hepcidin and ferritin was measured based on the principle of Sandwich-ELISA. The standard were diluted intubes, then (50 uL ) from each tube was added into the microplate and an empty well was left as blank control while in the sample wells, (40 μL) ofdilution buffer and (10 μL) from thesample was added, incubatedfor30 minutes and the wells were washed withthediluted washing buffer for five times. (50 μL) of HRP-Conjugate reagent was added into wells, incubated and washed again. For coloring (50 μL) of Chromogen A and (50 μL) of Chromogen B Solutions were added into each well and incubated at for 15 minutes (50 μL) stop solution was add into each well to terminate the reaction and within 15 minutes the absorbance was read at (450 nm) using a microtiter plate reader.

### Transferrin (Elabscience/USA) assay

The concentration of transferrin was measured based on the principle of Sandwich-ELISA. (100 μL) of diluted standard, sample and blank were addedinto wells and incubated for (90 min). The wells liquid were poured and (100 μL) of biotinylated solution was added into each well, incubated for 1 hour after that the wells contents were removed and rinsed three times with (350 μL) of wash buffer (200 μL) of HRP conjugate solution was added into all wells, incubated for 30 minutes then wells contents were poured an drinsed five times with (350 μL) of wash buffer. (90 μL) of substrate reagent was added, incubated for (15 minutes), then (50 μL) of stop solution was added into wells to stop enzymatic reaction. The optical density of the solution was reading at (450 nm) within 10 minutes with the microtiter plate reader.

### Iron (Biolabo/France) assay

The concentration of iron was measured by spectrophotometer. One mL of Reagent R1, and (200 μL) of specimen were mixed together and left for 3 minutes at room temperature, the A1 absorbance was recorded at (600 nm). One mL of working reagent and(200 μL) of specimen were mixed together and left for 5 minutes at room temperature, the A2 absorbance was recordedat (600 nm).

Result (μmol/L) =X Standard concentration.

### Testosterone (Demeditec Diagnostics/) assay

The ELISA method, which was used to measure the concentration of testosterone, relied on Competitive Binding principle. (25 μL) of standard, sample and control were dispensedinto microtiter wells, then (200 μL) of enzyme conjugate was added into each well, incubated for60 minutesafter that wells contents wereeliminated andthe wells wererinsed three times with (300 μL) of diluted wash. (200 μL) of substrate solution was added into wells, incubated for 15 minutes, then (100 μL) of stop solution was added to stop the enzymatic reaction. The solution optical density in the microtiter wells was reading at (450 nm) within 10 minutes with the microtiter plate reader.

### Progesterone (Monobind Inc. /USA) assay

The concentration of progesterone was measured based on the principle of Competitive Enzyme Immunoassay (type 7). (25 μL) of the standard, control and specimen were added into wells, then (50 μL) of enzyme reagent was added. After that (50 μL) of biotin reagent was added, incubated for 60 minutes. The contents of the microplate were poured, then (350 μL) of wash buffer was added and repeated two extra times.(100 μL) of substrate solution was added to wells, incubated for 20 minutes, then (50) μL of stop solution was added. Then within 15 minutes of adding stop solution the absorbance was recorded at (450 nm).

### Prolactin (Monobind Inc. /USA) assay

The level of prolactin was measured based on the principle of Sandwich ELISA method. (25 μL)of the standard, control and specimen was added into the wells, then (100 μL) of enzyme reagent was added and incubated about 60 minutes. The microplate contents were removed, then (350 μL) of wash buffer was added and repeated two further times. (100 μL) of substrate solution was added into the wells and incubated for 15 minutes. (50 μL) of stop solution was added and within 15 minutes the absorbance was measured at 450 nm.

### Estradiol (Bioactiva diagnostic/ Germany)

The concentration of estradiol was measured based on the principle of Competitive Enzyme Immunoassay ELISA method. (25 μL) of standard, samples and control were added into microplate wells, then (100 μL) of estradiol enzyme conjugate were added into each well and were incubated for 60 minutes. The wells contents were removed, and then were rinsed three times with (300 μL) diluted wash solution. (100 μL) of TMB reagentwas added andincubated for 30 minutes The reaction was stopped by adding 50 μL of stop solution and the absorbance was determinedat (450 nm) with the plate reader.

### Statistical analysis

The statistical analysis was conducted using SPSS version 23 (IBM Inc., Chicago, IL, USA). Descriptive statistics as mean and standard deviation (SD) for categorical data calculated. An association between variables assessed by t-test and Pearson’s correlation. ANOVA analysis was used to described the association between groups. A two-sided *P* value of less than 0.05 was considered statistically significant.

## Results

The current study recorded a significant decrease in the concentration of serum hepcidin, transferrin and estradiol, while data showed a significant increase in the concentration of ferritin, iron, progesterone and testosterone, but prolactin didn’t shown a significant difference in PCOS patients compared with control group, as showed in the Table 1.

**Table 1:** Comparison of study parameters among PCOS and control. *t-test = significant at P≤0.05

Variables	Control (n=45)	PCOS (n=45)
Hepcidin (μmol/L)	6.62±2.85	1.37±1.27*
Transferrin (g/L)	0.82±0.35	0.16±0.13*
Ferritin (μg/L)	41.97±26.35	155.02±82.23*
Iron (μmol/L)	81.81± 35.02	179.54± 51.8*
Estradiol (pmol/L)	109.97± 71.16	61.95±51.35*
Progesterone <br>(nmol/L)	11.42± 6.75	18.89± 18.8*
Testosterone <br>(nmol/L)	1.09± 0.8	1.30± 1.08*
Prolactin (μg/L)	12.11±6.19	12.74±4.46

Serum hepcidin showed a positive correlation with estradiol and an adverse correlation with progesterone and testosterone, while serum transferrin showed a positive correlation with estradiol and an adverse correlation with progesterone Table 2.

**Table 2:** Correlation of Hepcidin with sex hormones. **Pearson correlation is significant at P=0.01

Variables	Estradiol	progesterone	testosterone
Hepcidin	0.331**	-0.220-**	-0.259-**
Transferrin	0.181*	-0.167-*	-0.017-

A significant decrease in the level of serum hepcidin was observed in PCOS women with hirsutism and a significant decrease in hepcidin was observed in PCOS women with oligoamenorrhea, while a significant increase in hepcidin was observed in PCOS patients with menorrhagia, also non-significant variance was recorded in the serum hepcidin according to BMI, age, and infertility issues in PCOS patients compared, Table 3.

**Table 3:** Hepcidin level in PCOS patients depending on some factors. *ANOVA = significant at P≤0.05

Factor		Serum Hepcidin	
BMI	Normal /n=14	Overweight/n= 21	Obese/n=10
	8.23±6.99	8.84±8.59	4.77±2.46
Age	(19–29) /n=21	(30–40) /n=24	
	8.19±8.22	7.21±6.09	
Menstrual cycle	Normal /n=11	Oligoamenorrhea /n=18	Menorrhagia/n=16
	6.48±6.67	6.17±3.88	9.69±8.78*
Hirsutism	With /n=22	Non /n= 23	
	5.80±4.39	9.14±8.44*	

## Discussion

The current study showed a clear imbalance in the regulation of iron levels in PCOS patients, and this is consistent with many other studies. Hepcidin is a peptide hormone produced by the liver. It has a key role in iron metabolism [11]. Hepcidin level disturbance is associated with many diseases, it was observed that supplying testosterone to the laboratory rats of both sexes increases the levels of hemoglobin, iron, and erythropoietin and reduces the level of hepcidin, as it works to prevent the transcription of hepcidin by interacting with BMP/SMAD Pathway, also increases the binding of iron with RBCs [12]. Hepcidin controls the release of iron from their stores (hepatocyte) and from macrophage that circulates it, also hepcidin binds with ferroportin to stimulate iron degradation thus reduce its concentration in plasma, the most important regulators of hepcidin is iron concentration in the blood, inflammation, erythropoiesis and testosterone [13]. Currently, it has been found that progesterone in animals increases the gene expression of hepcidin and this has been observed in women who take progesterone to treat fertility problems as progesterone affects iron metabolism [14]. It has been observed that hepcidin decreases in the luteal phase and this decreasing is counteracted by elevated levels of progesterone and interleukins in this phase [15]. It has been observed that obesity in PCOS patients reduces iron level by increasing cytokines and oxidative stress then raising hepcidin, that prevents iron absorption from the intestine, thus anemia occurs as a risk factor for diabetes and the opposite can also occur where iron rises as a risk factor to type 2 diabetes, insulin resistance and cardiac disease [16]. The increasing in hematocrit and hemoglobin due to testosterone is associated with erythropoietin stimulation and with decreasing in hepcidin and ferritin levels, it was suggested that testosterone induces erythropoiesis by stimulating erythropoietin and also by consumption of iron in the erythropoiesis process [17]. High estrogen is associated with high iron, as estrogen lowers the level of hepcidin to improve the level of iron in the blood of menstruating women [18]. Hepcidin decreases iron absorption by binding to ferroportin causing iron breakdown and administrate the patient a dose of growth hormone or testosterone and reduces the level of hepcidin, also it was recorded gonadotropin-stimulated estrogen decreases the concentration of hepcidin-25 [19]. It was noticed that transcription of hepcidin was inhibited when hepatocytes were treated with 17 -estradiol in humans in order to increase iron consumption to recompense for iron lost during the menstrual cycle, also, this mechanism can increase iron storage in women who use contraceptives [20]. Lots of evidence shows that there is a relationship between iron metabolisms, diabetes and insulin resistance even with a slight increase in the level of iron; it has been observed that it is associated with high blood sugar; also ferritin elevates in PCOS patients who suffer from insulin resistance [21]. It was suggested that insulin resistance and high insulin levels, not amenorrhea or oligoamenorrhea, are responsible for the high level of ferritin and iron, especially in obese women with PCOS [22]. Transferrin plays an important role in the metabolism of iron and iron absorption by the cells, also acts as a growth factor [23]. An increase in the level of ferritin and insulin with a decrease in transferrin receptor was observed in obese PCOS women compared to the control group, where transferrin receptor can be relied on as an iron overload marker in PCOS patients [24]. Obesity and insulin are factors that raise the iron level in PCOS patients, which iron-oxidative stress governs the function of ovarian tissue, also ferritin and transferrin increases in obese PCOS patients with inverse correlation between ferritin and the of the ovaries size [25].

## Conclusions

There are clear imbalances in the level of iron and its serum regulating parameters in in PCOS women, and there is an effective correlation between iron status and sex hormones.

## Dodatak

### Authors’ Contribution

ADF: Conceptualization, Methodology, Formal analysis, Investigation, Resources, Data Curation, Writing - Original Draft, Writing - Review & Editing, Visualization, Project administration.

HHA: Methodology, Software, Resources, Data Curation, Writing - Original Draft, Writing - Review & Editing, Visualization.

### Ethical approval

The Al-Kunooze University College ethics committee reviewed and approved this study (No. 2020/10046).

### Consent of patients for publication

All patients provided written informed consent for the publication of their data.

### The exchange of research data

The data supporting the findings of this study are available on request from the corresponding author.

### Grant Support

None.

### Conflict of interest statement

All the authors declare that they have no conflict of interest in this work.
